# Dynamic changes of anterior segment in patients with different stages of primary angle-closure in both eyes and normal subjects

**DOI:** 10.1371/journal.pone.0177769

**Published:** 2017-05-18

**Authors:** Jialiu Lin, Zhonghao Wang, Chuchen Chung, Jianan Xu, Miaomiao Dai, Jingjing Huang

**Affiliations:** State Key Laboratory of Ophthalmology, Zhongshan Ophthalmic Center, Sun Yat-sen University, Guangzhou, China; University of Melbourne, AUSTRALIA

## Abstract

**Purpose:**

To compare changes in anterior segment parameters under light and dark (light-to-dark) conditions among eyes with chronic primary angle-closure glaucoma (CPACG), fellow eyes with confirmed or suspect primary angle-closure (PAC or PACS), and age-matched healthy eyes.

**Methods:**

Consecutive patients with CPACG in one eye and PAC/PACS in the fellow eye, as well as age-matched healthy subjects were recruited. Anterior segment optical coherence tomography measurements were conducted under light and dark conditions, and anterior chamber, lens, and iris parameters compared. Demographic and biometric factors associated with light-to-dark change in iris area were analyzed by linear regression.

**Results:**

Fifty-seven patients (mean age 59.6±8.9 years) and 30 normal subjects matched for age (60.6±9.3 years) and sex ratio were recruited. In regards to differences under light–to-dark conditions, angle opening distance at 500 μm (AOD500μm) and iris area during light-to-dark transition were smaller in CPACG eyes than fellow PACS/PAC eyes and normal eyes (*P*<0.017). Pupil diameter change was largest in normal eyes, and larger in PACS/PAC eyes than CPACG eyes (*P*<0.017). There was an average reduction of 0.145 mm^2^ in iris area for each millimeter of pupil diameter increase in CPACG eyes, 0.161 mm^2^ in fellow PAC/PACS eyes, and 0.165 mm^2^ in normal eyes. Larger iris curvature in the dark and diagnosis of PACG were significantly associated with less light-to-dark iris area changes.

**Conclusions:**

Dynamic changes in iris parameters with light-to-dark transition differed significantly among CPACG eyes, fellow PAC/PACS eyes, and normal eyes. Greater iris curvature under dark conditions was correlated with reduced light-to-dark change in iris area and pupil diameter, which may contribute to disease progression.

## Introduction

Primary angle-closure glaucoma (PACG) is a major cause of blindness globally, but especially in Asia [1,2]. Most ocular biometry studies of PACG have compared patients to normal subjects or acute primary angle closure (PAC) eyes to unaffected fellow eyes. Such studies have revealed much about the risk factors and pathogenesis of PACG. Shallow anterior chamber depth (ACD), short axial length (AL), greater lens thickness (LT), and larger lens vault (LV) are risk factors for PACG [3–5]. In addition, recent studies suggest that PAC pathogenesis depends not only on these static anatomic factors but also on different dynamic responses of the anterior chamber under light and dark conditions [6–10]. Anterior segment optical coherence tomography (AS-OCT) [6–10] and ultrasound biomicroscopy (UBM) [11] can provide high-definition images of the anterior segment *in vivo*, yielding reliable and repeatable quantitative measurements for assessing dynamic changes.

The classification for PAC includes primary angle closure suspect (PACS), PAC, and PACG [12]. The spectrum of disease could be regarded as: progression from PACS to PAC, then to PACG. However, the risk factors contributing to disease progression are not well-understood. Our previous study using UBM showed that a thin and anteriorly bowed iris may be associated with greater risk of progression from PAC/PACS to chronic PACG (CPACG) [13]. In the current study, we consider whether dynamic changes in anterior segment parameters are related to the asymmetry of disease development between the two eyes of CPACG patients. The objective was twofold: 1) to assess whether eyes at different stages of PAC exhibit different light-to-dark anterior segment changes; 2) to assess associations between these changes and demographic and ocular biometric parameters.

## Materials and methods

### Participants and ocular examinations

This is a cross-sectional comparative study. Consecutive patients diagnosed by two glaucoma specialists (JH & ZW) with CPACG in one eye and either confirmed or suspected primary angle-closure (PAC or PACS, respectively) in the fellow eye were recruited. Age and sex-matched healthy controls were recruited. The study was conducted at the Department of Glaucoma, Zhongshan Ophthalmic Center of Sun Yat-sen University (Guangzhou, China) from January 2014 to January 2015.

CPACG, PAC, PACS were defined according to International Society of Geographical and Epidemiological Ophthalmology (ISGEO) classifications [12]. Briefly, PACS is diagnosed when greater than 270° of the posterior trabecular meshwork is not visible on gonioscopy, while PAC is PACS associated with peripheral anterior synechiae (PAS) and/or elevated intraocular pressure (IOP) without glaucomatous damage of the optic nerve, and CPACG is PAC with evidence of optic nerve damage. PAC and CPACG eyes must not exhibit symptoms or signs of acute angle closure attack such as "glaukomflecken" or iris sphincter palsy. In this study, diagnosis of glaucomatous damage was based on 1) glaucomatous optic neuropathy, defined as a cup-to-disc ratio > 0.6 or asymmetry of >0.2 between the two eyes, 2) loss or thinning of the neuroretinal rim or retinal nerve fiber layer on clinical examination; 3) reliable and repeatable glaucomatous visual field defect, defined by SITA standard 30–2 or 24–2 Humphery perimetry (Carl Zeiss, Dublin, CA, USA) as two or more contiguous points with a sensitivity pattern deviation at *P*<0.01 or three or more contiguous points with a sensitivity pattern deviation at *P*<0.05 in the superior or inferior arcuate areas (compared with that of perimeter-defined age-matched control subjects), or a 10-dB difference across the nasal horizontal midline at two or more adjacent locations and an abnormal glaucoma hemifield test (false positive/negative rate <15%, fixation loss <10%) [14].

Inclusion criteria for normal subjects were 1) IOP < 21 mmHg by Goldmann applanation tonometry, 2) wide anterior chamber angle determined with Shaffer classification by gonioscopy, 3) normal optic nerve appearance by dilated stereoscopic examination, 4) eligible visual field within normal range by Humphrey perimetry, 5) refractive ranges from +1D to -3D, 6) no medical or family history of retinal diseases or glaucoma, 7) no medical or family history of diabetes mellitus, 8) free of topical medications except artificial tears and anti-cataract eye drops, and free of systemic medications such as diuretics and high osmotic agents, and 9) no prior intraocular or laser surgery on either eye. The age range of included normal subjects was 50−80 years old.

The exclusion criteria for patients were 1) secondary angle closure such as neovascular, uveitic, or traumatic glaucoma, 2) previous laser or intraocular surgery on either eye, 3) subluxated lens or intumescent cataract, 4) uveal effusion or retinal detachment, 5) history or current use of topical or systemic cholinergic agents within 7 days that could affect iris or pupil size, and 6) AL less than 19 mm in either eye.

The study was conducted in accordance with the tenets of the Declaration of Helsinki and approved by the Institutional Review Board of Zhongshan Ophthalmic Center. Written informed consent was obtained from all patients and controls. All subjects underwent detailed ocular examinations, including best-corrected visual acuity by Snellen chart, slit-lamp examination, stereoscopic optic disc examination with a 90-diopter lens, and IOP measurement by Goldmann applanation tonometry. Gonioscopy was performed in the dark using a Goldmann one-mirror lens at high magnification. The extent of PAS in each eye was assessed by both glaucoma specialists (JH & ZW). In all eyes except one, the difference between the two assessments was less than one clock hour. If a discrepancy occurred, a second examination by the two glaucoma specialists was performed and the results averaged. AL was measured by A-scan ultrasonic biometry (Model KN-3000A; Quantel Co, Ltd., France).

### Anterior segment optical coherence tomography (AS-OCT)

AS-OCT (Visante 1.0; Carl Zeiss Meditec, CA.) examinations and measurements were performed by the same trained physician (LJ) who was masked to the clinical data. IOP of the examined patients was controlled below 30 mmHg in both eyes using anti-glaucoma eye drops when indicated to minimize corneal edema, which may affect AS-OCT measurements of the anterior segment. Topical cholinergic agents were discontinued for at least 7 days prior to AS-OCT examinations. No prostaglandin analogs or osmotic agents were used as they may affect anterior segment measurements. AS-OCT examinations were first performed with subjects sitting in a bright room (illumination 750 to 800 Lux, Model TES-1339; TES Electrical Corp.) and then in a dark room (illumination 0.2 to 1 Lux). The subjects adapted to the dark for at least 5 minutes prior to examination. During AS-OCT scanning, an internal fixation target was used with the subjects’ refractive correction to perform the measurements in an unaccommodated state. Horizontal standard anterior segment single-scan mode (0° to 180°) was used for perpendicular scans centered over the pupil and repeated three times. Aligment that results in a central corneal reflex ensures good repeatability as recommended by the manufacturer. After image capture, the best image was selected and stored in the system for subsequent ocular biometric measurements.

**Fig 1:**
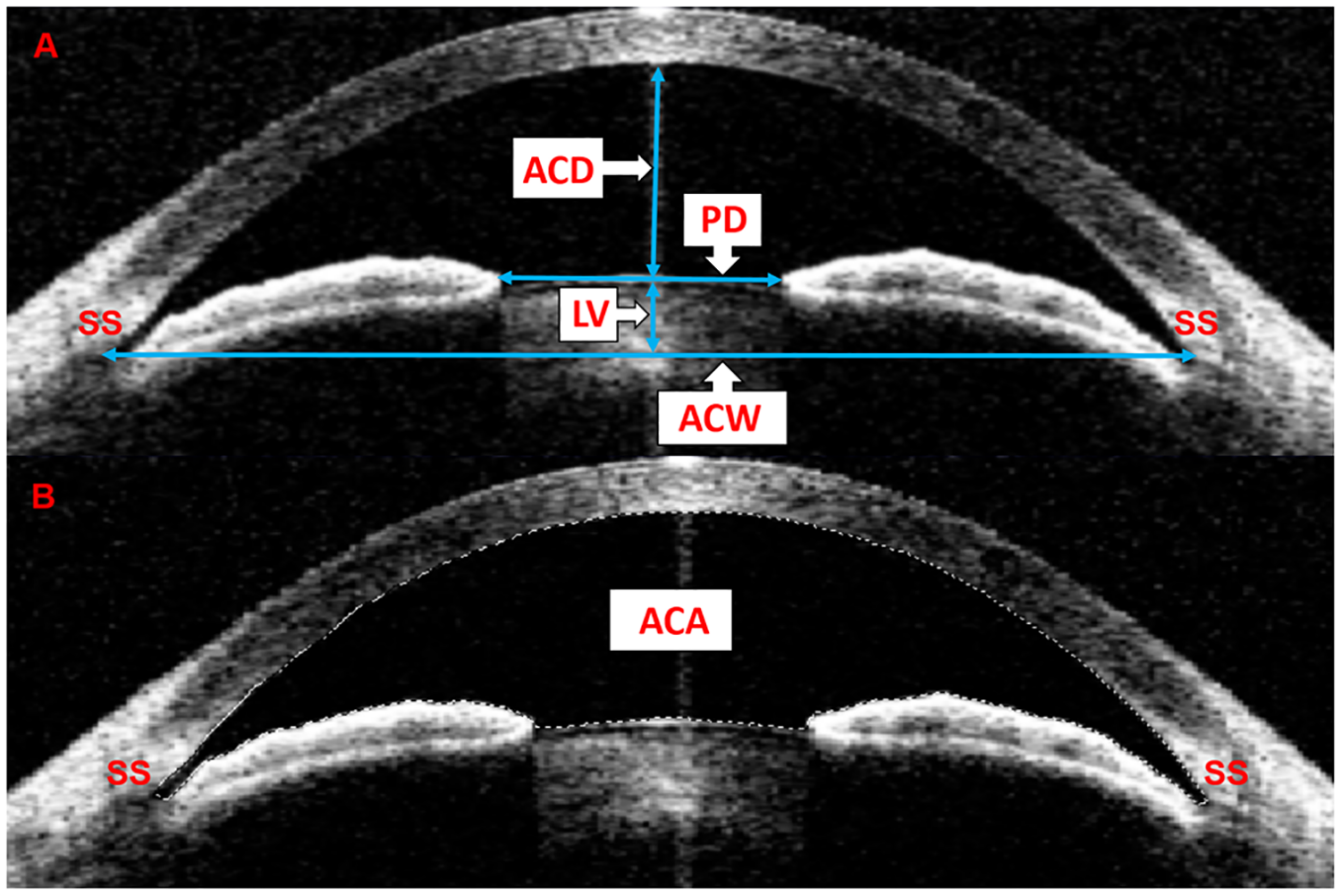
Determinations of ACD, ACW, ACA, PD and LV by AS-OCT. A: Anterior chamber depth (ACD) was defined as the axial distance from the corneal endothelium to the anterior lens surface. Anterior chamber width (ACW) was defined as the distance between the two scleral spurs. Pupil diameter (PD) was the distance between the pupil edges of the iris. Lens vault (LV) was defined as the perpendicular distance between the anterior pole of the crystalline lens and the horizontal line connecting the two scleral spurs. B: Anterior chamber area (ACA) was defined as the cross-sectional area of the anterior chamber bordered by the posterior surface of the cornea, the anterior surface of the iris and the anterior surface of the lens within the pupil.

**Fig 2:**
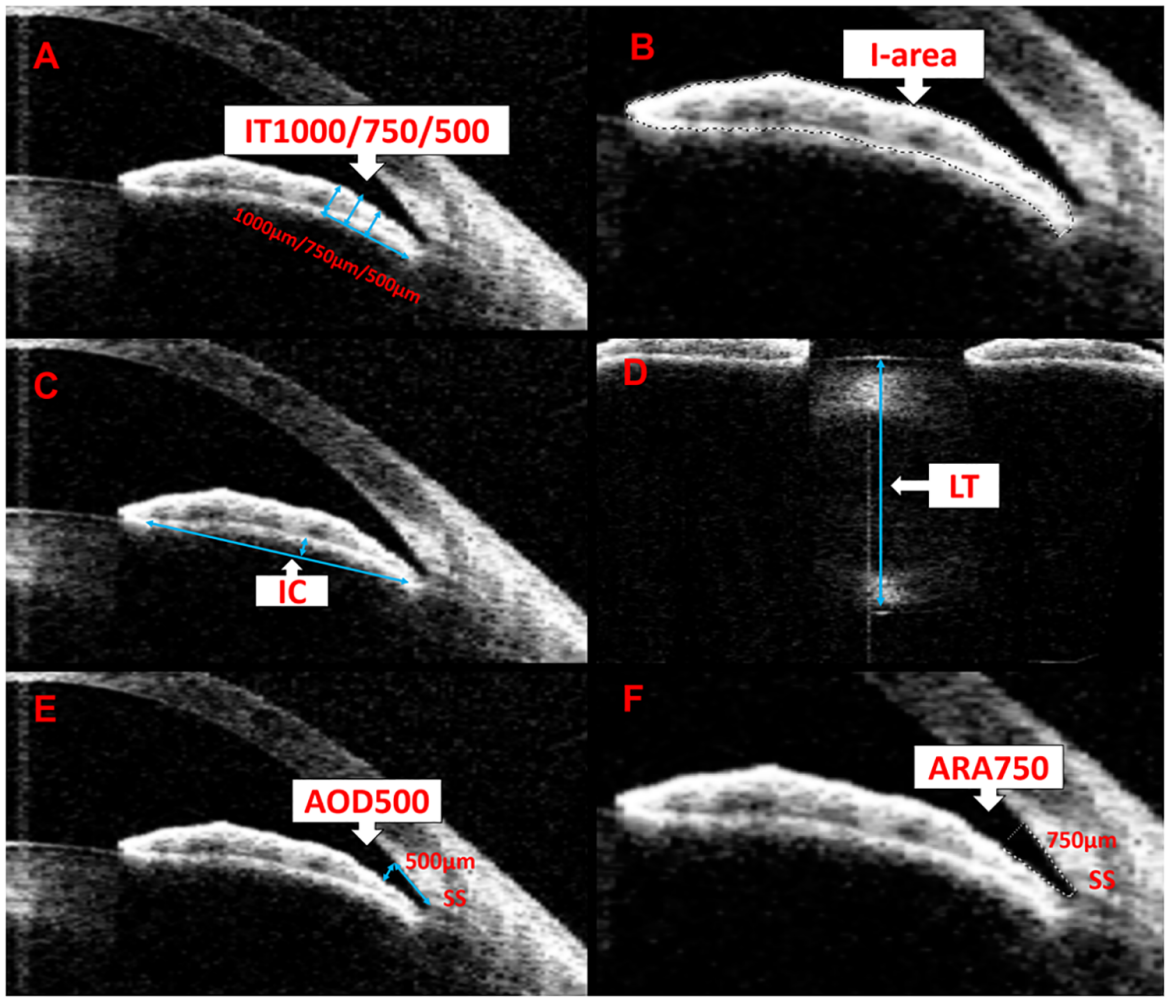
Determinations of AOD500μm, IT500μm/750μm/1000μm, IC, LT, ARA750μm, and I-area by AS-OCT. A: Iris thickness at 500, 750, and 1000 μm (IT500μm, IT750μm, and IT1000μm, respectively) were defined as the perpendicular distance from the point at iris pigment epithelium anterior to the iris surface at 500, 750, and 1000 μm from the iris root, respectively. B: Iris area (I-area) was defined as the cross-sectional area of the iris. C: Iris curvature (IC) was defined as the maximum perpendicular distance between the iris pigment epithelium and the line connecting the most peripheral to the most central point of the epithelium. D: Lens thickness (LT) was defined as the maximum perpendicular distance between the anterior and posterior poles of the lens. E: Angle opening distance at 500 μm (AOD500μm) was defined as the perpendicular distance from the point anterior to the scleral spur to the anterior iris surface at 500 μm from the scleral spur. F: Angle recess area at 750 μm (ARA750μm) was defined as the enclosed triangular area demarcated by the anterior iris surface, trabecular meshwork, and corneal endothelium to a distance of 750 μm from the scleral spur.

AS-OCT linear parameters were measured as described previously using the caliper in the instrument [5,6]. Briefly, ACD was defined as the axial distance from the corneal endothelium to the anterior lens surface (Fig 1A). Anterior chamber width (ACW) was the distance between the 2 scleral spurs (Fig 1A). Pupil diameter (PD) was the distance between the pupil edges of the iris (Fig 1A). LV was defined as the perpendicular distance between the anterior pole of the crystalline lens and the horizontal line connecting the two scleral spurs (Fig 1A). Iris thickness (the perpendicular distance from iris pigment epithelium to the anterior iris surface) was measured at 500, 750, and 1000 μm from the iris root (IT500μm, IT750μm, and IT1000μm, respectively) (Fig 2A). Iris curvature (IC) was defined as the maximum perpendicular distance between the iris pigment epithelium and the line connecting the most peripheral to the most central point of the epithelium (Fig 2C). LT was defined as the maximum perpendicular distance between the anterior and posterior poles of the lens (Fig 2D). Angle opening distance (defined as the perpendicular distance from the point anterior to the scleral spur to the anterior iris surface) was measured at 500 μm from the scleral spur (AOD500μm) (Fig 2E). All area measurements were performed using the “Magnetic Lasso Tool” in Photoshop (Adobe Photoshop CS4, Adobe Systems Inc, CA, USA) as described in our previous study [15]. Anterior chamber area (ACA) was defined as the cross-sectional area of the anterior chamber bordered by the posterior surface of the cornea, the anterior surface of the iris, and the anterior surface of the lens within the pupil (Fig 1B). Iris area (I-area) was the cross-sectional area of the iris (Fig 2B). Angle recess area (ARA750μm) was defined as the enclosed triangular area demarcated by the anterior iris surface, the trabecular meshwork, and the corneal endothelium at 750 μm from the scleral spur (Fig 2F). Every parameter was measured 3 times and the average value recorded. Both the nasal and temporal sides of the structures were measured then averaged.

All parameters were measured by a single physician (LJ), so we also investigated intraobserver reproducibility in 30 glaucoma eyes and 30 normal eyes by repeated measurements in two sessions at an interval of two weeks.

### Statistical analysis

Statistical analyses were performed using SPSS software version 18.0 (SPSS, Inc., Chicago, IL). The means and standard deviations of the above parameters and changes from light to dark were calculated. Mean values between the CPACG eye and the fellow eye were compared by paired *t* test. Mean values of parametric data were compared among subgroups by independent samples *t* test. Gender related differences among the diagnostic groups by *chi-square* test. Variables without normal distribution, including the optic disc ratio and the extent of PAS, were compared by *Wilcoxon* rank test for related samples or *Mann-Whitney U* test for independent samples as appropriate. For all tests among multiple groups, *P*<0.017 (0.05/3) was considered significant. Intraobserver correlation coefficient (ICC) and 95% limits of agreement were used to evaluate the reproducibility of AS-OCT measurements of linear parameters and area parameters. Pearson’s correlation coefficient was used to assess the relationship between light-to-dark changes of PD and I-area with *P*<0.05 considered significant.

Univariate regression was conducted to evaluate age, sex, IOP, AL, and anterior segment parameters (ACA, LV, LT, IT500, and PD in dark condition) as predictors of dynamic changes between light and dark conditions. Anterior segment parameters that were significant at a level of *P*<0.1 were included in a multiple linear regression model adjusting for sex, age, IOP, AL, extent of PAS, and diagnostic group. The diagnostic group was considered an unordered categorical variable that did not conform to the requirements of an independent variable in regression analysis, so we set two dummy variables. As it was inappropriate to pool the data of both two eyes from a single subject in the regression analysis, we included only the left eyes of CPACG patients (including 31 PACG eyes and 26 PAC/PACS eyes) and normal controls.

## Results

A total of 57 patients with CPACG (34 with PAC and 23 with PACS in fellow eyes) and 30 healthy subjects met eligibility criteria. All subjects were of Chinese ethnicity. There was no difference in age and sex ratio between CPACG patients and normal subjects (Table 1). Normal eyes and PACS/PAC eyes had significantly better visual acuity and visual field parameters than CPACG eyes. Baseline IOP and cup-to-disc ratio were higher in CPACG eyes than in the other two groups. PAS was more extensive in CPACG eyes than in PACS/PAC eyes. Axial length was shorter in CPACG eyes and fellow PACS/PAC eyes than in normal eyes. Intraobserver reproducibility was acceptable for all AS-OCT measurements (S1 Table).

**Table 1:** Demographic and biometric characteristics of CPACG patients (CPACG eyes *vs* fellow PAC/PACS eyes) and normal subjects.

	CPACG patients		Normal subjects	*P* Value		
	CPACG eyes (mean±SD) (n = 57)	PAC/PACS eyes (mean±SD) (n = 57)	Normal eyes (mean±SD) (n = 30)	CPACG eyes *vs* PAC/PACS eyes*	Normal eyes *vs* CPACG eyes†	Normal eyes *vs* PAC/PACS eyes†
**Female (%)**	33 (57.9%)		17 (56.7%)	/	0.912♯	0.912♯
**Age (year)**	59.6±8.9		60.6±9.3	/	0.629	0.629
**Right eye (%)**	26 (45.6%)	31 (54.4%)	16 (53.3%)	0.349	0.493	0.925
**Baseline IOP (mmHg)**	26.8±8.7	15.0±4.9	14.1±3.1	**<0.001**	**<0.001**	0.438
**LogMAR VA**	0.66±0.81	0.11±0.16	0.19±0.29	**<0.001**	**<0.001**	0.181
**C/D ratio**	0.81±0.18	0.41±0.11	0.37±0.11	**<0.001**¢	**<0.001**§	0.064§
**Extent of PAS (clock-hours)**	8.5±2.5	2.1±2.4	0.00±0.00	**<0.001**¢	**<0.001**§	**<0.001**§
**MD of VF (dB)**	-18.59±10.43	-4.24±2.73	-4.19±5.34	**<0.001**	**0.001**	0.841
**PSD of VF (dB)**	7.49±3.09	2.42±4.49	2.27±1.30	**<0.001**	**<0.001**	0.861
**AL (mm)**	22.60±0.79	22.50±0.77	23.69±1.11	0.474	**0.001**	**<0.001**
**Spherical equivalent (D)**	0.49±1.76	0.67±1.41	-0.17±1.01	0.467	0.535	0.535

* Paired *t*-test

^**†**^ Independent *t*-test

^**♯**^ *chi-square* test

^**¢**^ *Wilcoxon rank* test

^**§**^ *Mann-Whitney U* test

**CPACG:** Chronic primary angle-closure glaucoma

**PAC:** Primary angle-closure

**PACS:** Primary angle-closure suspect

**Female (%):** The percent of women patients

**Right eye (%):** The percent of right eyes

**IOP:** Intraocular pressure

**VA:** Visual acuity

**C/D:** Cup- to-disc ratio

**PAS:** Peripheral anterior synechiae

**MD:** Mean deviation

**VF:** Visual field

**PSD:** Pattern standard deviation

**AL:** Axial length

The ACD and ACA were smallest in CPACG eyes, and were smaller in fellow PACS/PAC eyes than in normal eyes under both light and dark conditions. Similarly, AOD500μm and ARA750μm were smallest in CPACG eyes, followed by fellow PACS/PAC eyes under light conditions. LV and LT were much larger in CPACG eyes and fellow PACS/PAC eyes than in normal eyes under both light and dark conditions, while there were no differences in the LVs and LTs between CPACG and fellow PACS/PAC eyes under either condition (Table 2).

**Table 2:** Anterior segment parameters under light and dark conditions and light-dark changes (Δ) in CPACG patients (CPACG eyes *vs* fellow PAC/PACS eyes) and normal subjects.

		CPACG patients		Normal subjects	*P* Value		
		CPACG eyes (mean±SD)	PAC/PACS eyes (mean±SD)	Normal eyes (mean±SD)	CPACG eyes *vs* PAC/PACS eyes*	Normal eyes *vs* CPACG eyes†	Normal eyes *vs* PAC/PACS eyes†
**ACD (mm)**	**Light**	2.008±0.228	2.079±0.213	2.533±0.329	**0.002**	**<0.001**	**<0.001**
	**Dark**	2.019±0.239	2.085±0.218	2.540±0.332	**0.005**	**<0.001**	**<0.001**
	**ΔACD**	-0.010±0.043	-0.007±0.033	-0.008±0.028	0.570	0.776	0.895
**ACW (mm)**	**Light**	11.292±0.436	11.353±0.405	11.561±0.446	0.058	**0.008**	0.030
	**Dark**	11.218±0.409	11.284±0.442	11.520±0.418	0.089	**0.002**	0.018
	**ΔACW**	0.074±0.273	0.069±0.206	0.042±0.159	0.903	0.488	0.526
**ACA (mm**^**2**^**)**	**Light**	13.248±2.114	13.869±2.064	18.084±3.239	**0.001**	**<0.001**	**<0.001**
	**Dark**	13.857±2.346	14.668±2.298	19.195±3.421	**<0.001**	**<0.001**	**<0.001**
	**ΔACA**	-0.610±0.811	-0.800±0.584	-1.101±1.323	0.042	0.035	0.143
**AOD500μm(mm)**	**Light**	0.105±0.058	0.151±0.069	0.359±0.080	**<0.001**	**<0.001**	**<0.001**
	**Dark**	0.066±0.062	0.079±0.059	0.273±0.093	0.106	**<0.001**	**<0.001**
	**ΔAOD500μm**	0.039±0.049	0.073±0.062	0.086±0.058	**0.001**	**<0.001**	0.335
**ARA750μm (mm**^**2**^**)**	**Light**	0.068±0.039	0.095±0.048	0.217±0.044	**<0.001**	**<0.001**	**<0.001**
	**Dark**	0.043±0.037	0.052±0.041	0.179±0.063	0.099	**<0.001**	**<0.001**
	**ΔARA750μm**	0.025±0.029	0.043±0.041	0.039±0.044	**0.005**	0.134	0.646
**LV (mm)**	**Light**	0.800±0.226	0.768±0.223	0.432±0.233	0.206	**<0.001**	**0.001**
	**Dark**	0.774±0.236	0.736±0.223	0.393±0.243	0.201	**<0.001**	**<0.001**
	**ΔLV**	0.026±0.105	0.031±0.101	0.038±0.075	0.766	0.557	0.729
**LT (mm)**	**Light**	5.041±0.355	5.007±0.390	4.780±0.399	0.475	**0.002**	**0.012**
	**Dark**	5.033±0.347	5.029±0.438	4.770±0.375	0.942	**0.002**	**0.007**
	**ΔLT**	0.008±0.195	-0.023±0.207	0.010±0.101	0.447	0.963	0.419

* Paired *t*-test

^**†**^ Independent *t*-test

**CPACG:** Chronic primary angle-closure glaucoma

**PAC:** Primary angle-closure

**PACS:** Primary angle-closure suspect

**Light:** Parameters in light condition

**Dark:** Parameters in dark condition

**ACD:** Anterior chamber depth

**ACW:** Anterior chamber width

**ACA:** Anterior chamber cross-sectional area

**AOD500μm:** Angle opening distance 500μm from the scleral spur

**ARA750μm:** Angle recess area 750μm from the scleral spur

**LV:** Lens vault

**LT:** Lens thickness

**Δ:** Changes from light to dark

**Fig 3:**
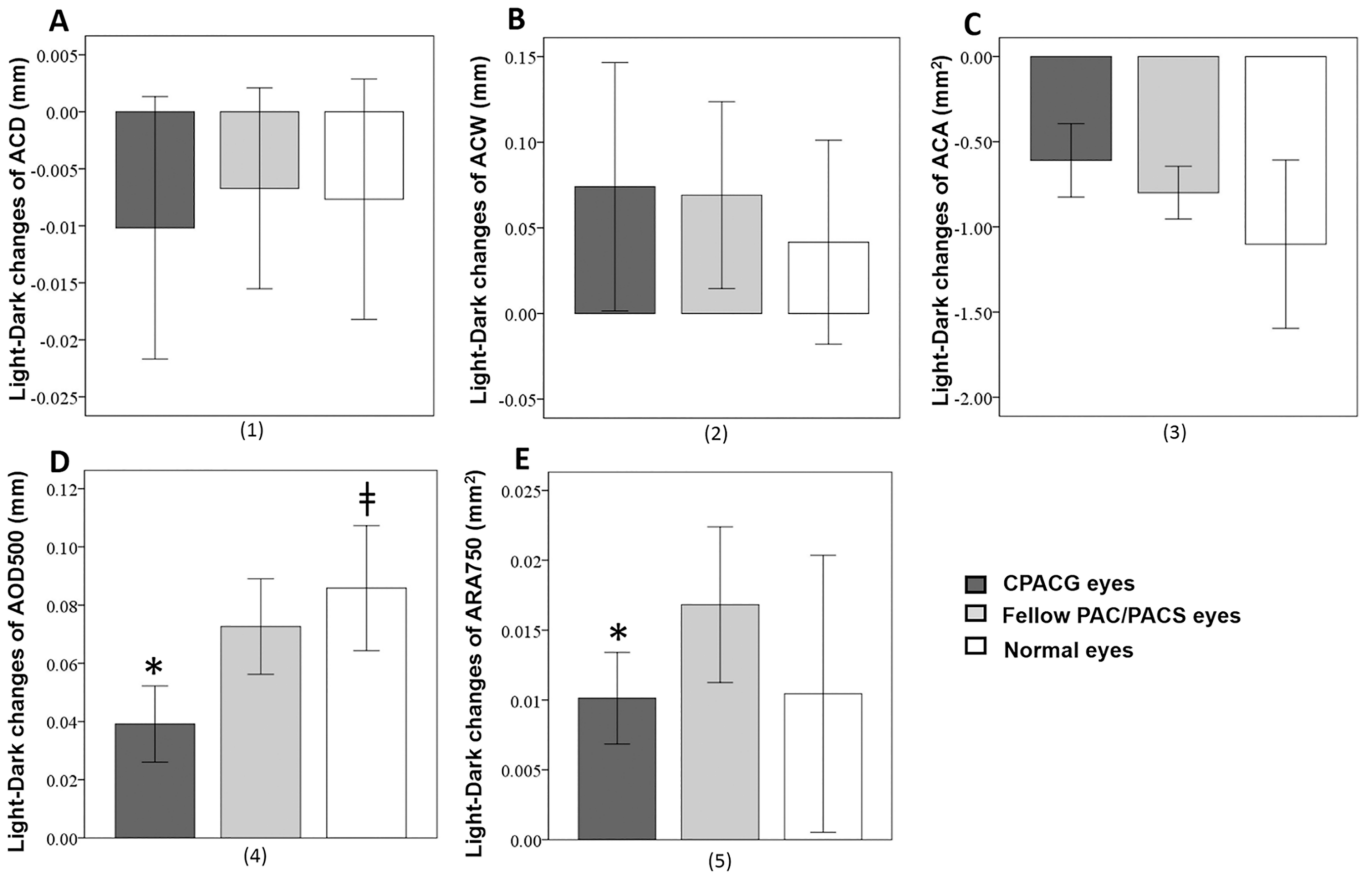
Light-to-dark changes of anterior chamber parameters in CPACG eyes, fellow PAC/PACS eyes, and normal eyes. A: There was no difference in light-to-dark changes of ACDs among the three groups. B: There was no difference in light-to-dark changes of ACWs among the three groups. C: There was no difference in light-to-dark changes of ACAs among the three groups. D: The light-to-dark changes of AOD500μm in CPACG eyes were smaller than those in their fellow PACS/PAC eyes and normal eyes. There was no difference in the light-to-dark changes of AOD500μm between PACS/PAC eyes and normal eyes. E: The light-to-dark changes of ARA750μm in CPACG eyes were smaller than those in their fellow PACS/PAC eyes. There was no difference in the light-to-dark changes of ARA750μm between PACS/PAC eyes and normal eyes. CPACG: chronic primary angle closure glaucoma; PAC: primary angle closure; PACS: primary angle closure suspect; ACA: anterior chamber area; AOD500μm: angle opening distance 500 μm from the scleral spur; ARA750μm: angle recess area 750 μm from the scleral spur; ACD: anterior chamber depth; ACW: anterior chamber width; *: significant difference between PACG eyes and fellow eyes (*P*<0.017); ‡: significant difference between PACG eyes and normal eyes (*P*<0.017); Error bars: 95% confidence interval (95% CI).

**Fig 4:**
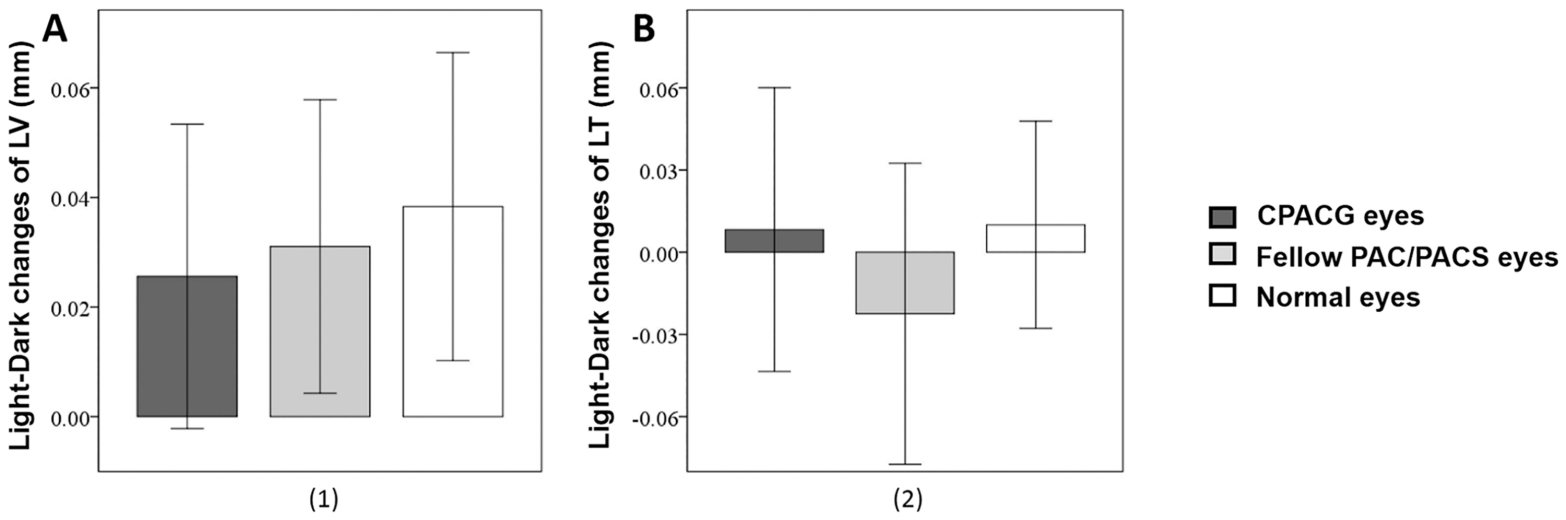
Light-to-dark changes of lens parameters in CPACG eyes, fellow PAC/PACS eyes, and normal eyes. A: There was no difference in light-to-dark change of LVs among the three groups. B: There was no difference in light-to-dark change of LTs among the three groups. CPACG: chronic primary angle closure glaucoma; PAC: primary angle closure; PACS: primary angle closure suspect; LV: lens vault; LT: lens thickness; Error bars: 95% confidence interval (95% CI).

The change in AOD500μm from light to dark was smaller in CPACG eyes than fellow PACS/PAC eyes and normal eyes (Fig 3D). Similarly, the change in ARA750μm between light to dark was smaller in CPACG eyes than fellow PACS/PAC eyes (Fig 3E). There were no differences in light-to-dark AOD500μm and ARA750μm changes between PACS/PAC eyes and normal eyes (Fig 3D and 3E). There was no light-to-dark difference in ACD, ACW, ACA, LV, or LT among the three groups (Figs 3A, 3B, 3C, 4A and 4B).

**Fig 5:**
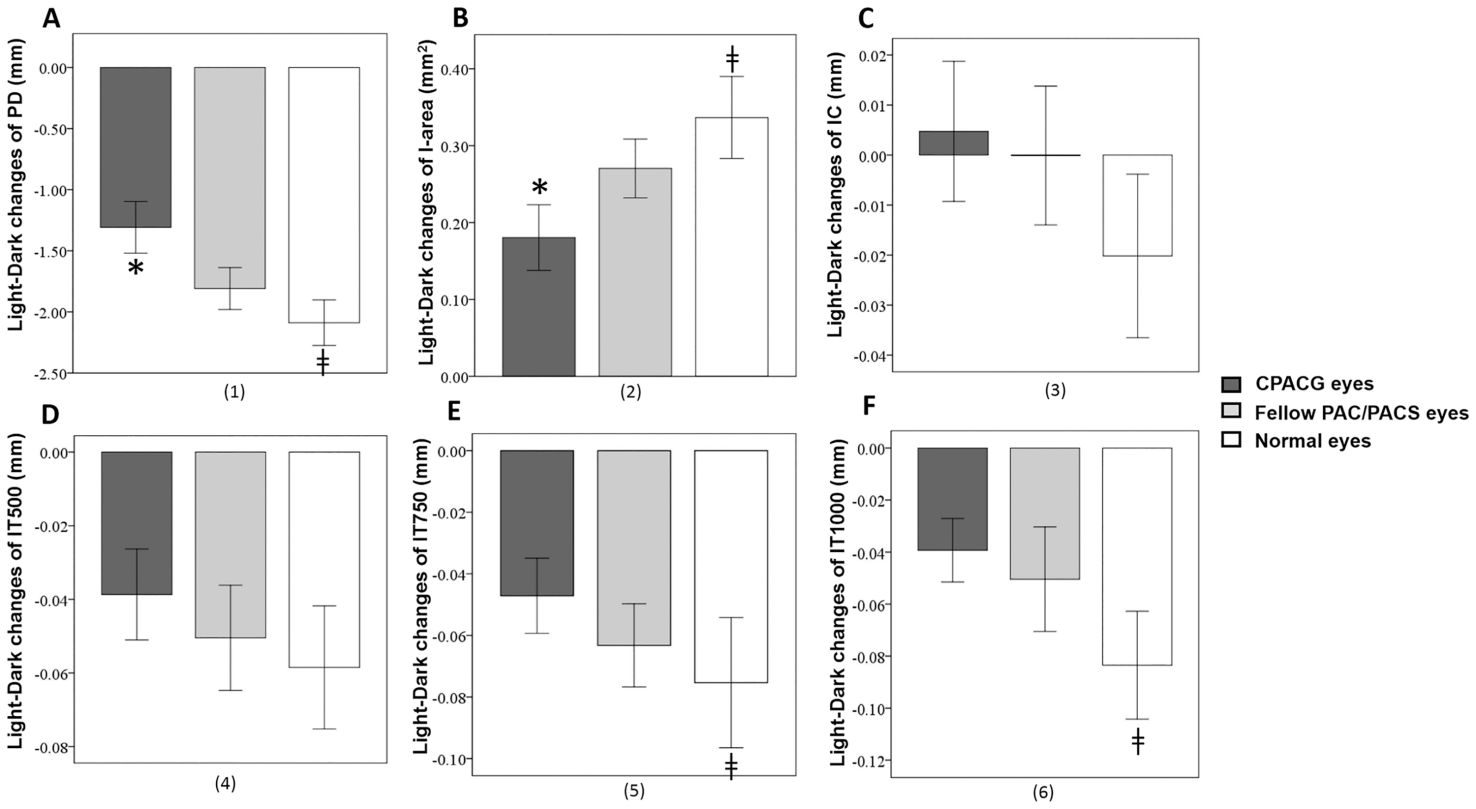
Light-to-dark changes of iris parameters in CPACG eyes, fellow PAC/PACS eyes, and normal eyes. A: The light-to-dark changes of PDs were largest in normal eyes and least in CPACG eyes. B: The light-to-dark changes of I-areas were largest in normal eyes and least in CPACG eyes. C: There was no significant difference in iris curvature changes in response to light among normal eyes, PACS/PAC eyes, and CPACG eyes. D: There was no significant difference in the light-to-dark changes of IT500μm among normal eyes, PACS/PAC eyes, and CPACG eyes. E: The light-to-dark changes of IT750μm were smaller in CPACG eyes than in normal eyes. There was no significant difference between PACS/PAC eyes and CPACG eyes in the iris thickness. F: The light-to-dark changes of IT1000μm were smaller in CPACG eyes than in normal eyes. There was no significant difference between PACS/PAC eyes and CPACG eyes in the iris thickness. CPACG: chronic primary angle closure glaucoma; PAC: primary angle closure; PACS: primary angle closure suspect; PD: pupil diameter; I-area: iris area; IC: iris curvature; IT500μm/IT750μm/IT1000μm: iris thicknesses 500 μm/750 μm/1000 μm from the iris root. *: significant difference between PACG eyes and fellow eyes (*P*<0.017); †: significant difference between fellow PAC/PACS eyes and normal eyes (*P*<0.017); ‡: significant difference between PACG eyes and normal eyes (*P*<0.017); Error bars: 95% confidence interval (95% CI).

As shown in Table 3, PD was larger in CPACG eyes than normal eyes in the light condition but did not differ among groups in the dark condition. Conversely, I-area was smaller in CPACG eyes than fellow PACS/PAC eyes and normal eyes in the light condition, but did not differ among groups in the dark condition. Therefore, light-to-dark PD and I-area changes were largest in normal eyes and smallest in CPACG eyes (Fig 5A and 5B).

**Table 3:** Iris parameters under light/dark conditions and changes (Δ) in CPACG patients (CPACG eyes *vs* fellow PAC/PACS eyes) and normal subjects.

		CPACG patients		Normal subjects	*P* Value		
		CPACG eyes (mean±SD)	PAC/PACS eyes (mean±SD)	Normal eyes (mean±SD)	CPACG eyes *vs* PAC/PACS eyes*	Normal eyes *vs* CPACG eyes†	Normal eyes *vs* PAC/PACS eyes†
**PD (mm)**	**Light**	3.025±0.756	2.833±0.410	2.638±0.398	0.028	**0.003**	0.038
	**Dark**	4.334±1.039	4.641±0.783	4.727±0.685	0.018	0.041	0.353
	**ΔPD**	-1.308±0.773	-1.809±0.628	-2.088±0.500	**<0.001**	**<0.001**	0.028
**I-area (mm**^**2**^**)**	**Light**	1.777±0.252	1.889±0.200	1.970±0.132	**<0.001**	**<0.001**	0.029
	**Dark**	1.596±0.224	1.620±0.166	1.633±0.136	0.366	0.351	0.661
	**ΔI-area**	0.180±0.156	0.270±0.140	0.337±0.143	**<0.001**	**<0.001**	0.045
**ΔI-area/ΔPD**		0.145±0.211	0.161±0.104	0.165±0.068	0.607	0.530	0.857
**IT500μm (mm)**	**Light**	0.403±0.068	0.417±0.060	0.409±0.064	0.078	0.677	0.584
	**Dark**	0.441±0.080	0.467±0.068	0.468±0.066	**0.008**	0.128	0.975
	**ΔIT500μm**	-0.039±0.047	-0.050±0.054	-0.059±0.044	0.241	0.059	0.485
**IT750μm (mm)**	**Light**	0.411±0.075	0.418±0.056	0.412±0.065	0.346	0.937	0.675
	**Dark**	0.458±0.083	0.481±0.069	0.488±0.070	**0.011**	0.099	0.681
	**ΔIT750μm**	-0.047±0.046	-0.063±0.051	-0.075±0.057	0.065	**0.014**	0.314
**IT1000μm (mm)**	**Light**	0.424±0.074	0.429±0.074	0.420±0.056	0.523	0.793	0.515
	**Dark**	0.464±0.079	0.479±0.094	0.504±0.057	0.173	**0.016**	0.194
	**ΔIT1000μm**	-0.039±0.046	-0.050±0.076	-0.084±0.056	0.290	**<0.001**	0.038
**IC(mm)**	**Light**	0.255±0.073	0.263±0.069	0.189±0.060	0.359	**<0.001**	**<0.001**
	**Dark**	0.251±0.068	0.263±0.062	0.209±0.048	0.056	**0.004**	**<0.001**
	**ΔIC**	0.005±0.053	-0.000±0.053	-0.020±0.044	0.596	0.030	0.076

* Paired *t*-test

^**†**^ Independent *t*-test

**CPACG:** Chronic primary angle-closure glaucoma

**PAC:** Primary angle-closure

**PACS:** Primary angle-closure suspect

**Light:** Parameters in light condition

**Dark:** Parameters in dark condition

**Δ:** Change from light to dark

**PD**: Pupil diameter

**IT500μm/ IT750μm/ IT1000μm:** Iris thickness 500 μm/750 μm/1000 μm from the iris root

**IC:** Iris curvature

**I-area:** Iris cross-sectional area

No differences were found in iris thicknesses among the three groups under the light condition (Table 3). In the dark, IT500μm and IT750μm were smaller in CPACG eyes than in PACS/PAC eyes, while IT1000μm was smaller in CPACG eyes than normal eyes. Therefore, changes in the iris thickness 500 and 1000μm from the iris root in response to light were smaller in CPACG eyes than normal eyes (Fig 5E and 5F). There were no significant differences in ΔIC among normal, PACS/PAC, and CPACG eyes in response to light (Table 3, Fig 5C), while IC was smaller in normal eyes under both light and dark conditions compared to PACS/PAC and CPACG eyes (Table 3).

**Fig 6:**
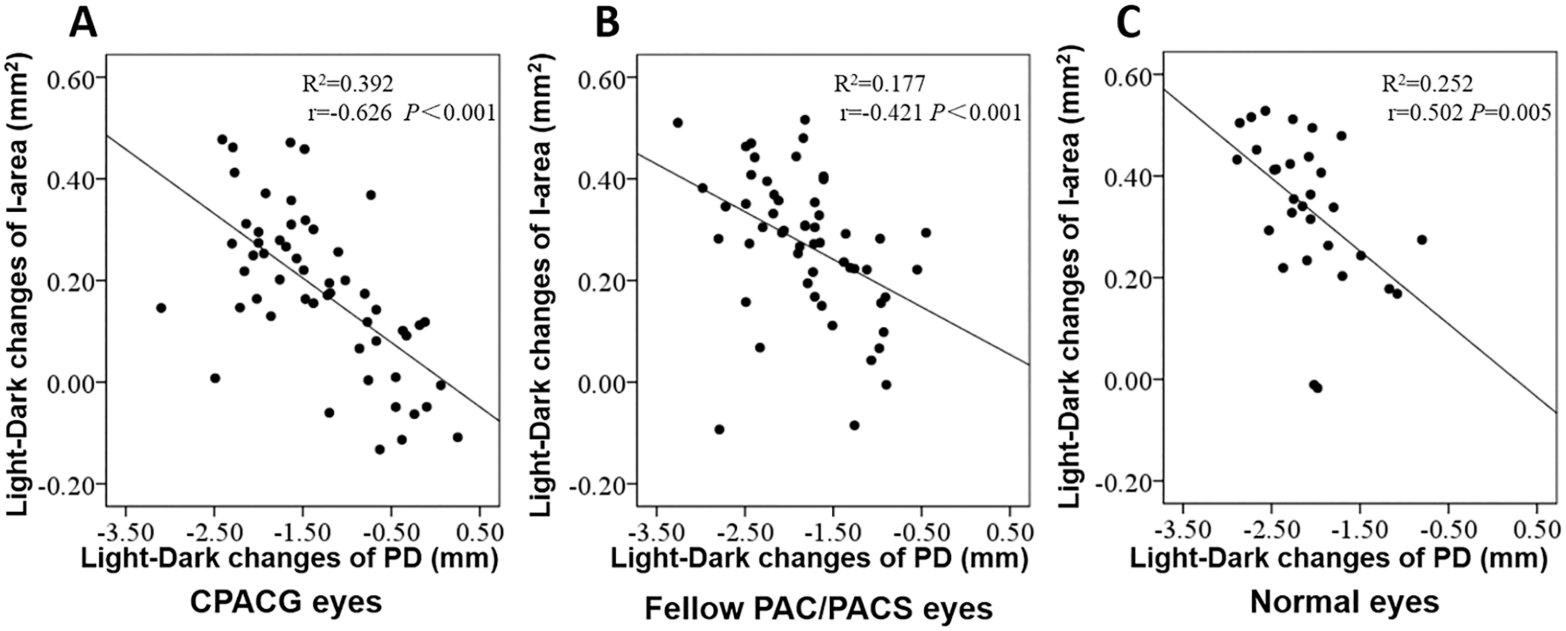
Relationship between mean PD change and I-area change after physiological mydriasis in CPACG eyes, fellow PAC/PACS eyes, and normal eyes. The I-area decreased with increasing pupil size in all three groups. A: For CPACG eyes, the linear regression equation of the scatter plot was y = 0.015–0.127x. B: For fellow PAC/PACS eyes, the linear regression equation of the scatter plot was y = 0.101–0.094x. C: For normal eyes, the linear regression equation of the scatter plot was y = 0.037–0.144x. CPACG: chronic primary angle closure glaucoma; PAC: primary angle closure; PACS: primary angle closure suspect; PD: pupil diameter; I-area: iris area.

The relationship between mean PD changes and I-area changes after physiological mydriasis is illustrated in Fig 6. The I-area decreased with increasing pupil size in all three groups (*r* = -0.660 in CPACG eyes, *r* = -0.487 in fellow PAC/PACS eyes, *r* = -0.502 in normal eyes, all *P*<0.05). For each millimeter increase in PD, there was an estimated 0.145 mm^2^ iris area loss in CPACG eyes, 0.161 mm^2^ in fellow PAC/PACS eyes, and 0.165 mm^2^ in normal eyes (Table 3). There was no significant difference in I-area change with pupil dynamic change among groups (Table 3).

Univariate and multiple linear regression analyseis revealed larger IC in the dark and PACG diagnosis as significant predictors of smaller light-to-dark change in I-area (Table 4).

**Table 4:** Factors in dark condition associated with light-to-dark changes in I-area by univariate and multiple regression between the left eyes of CPACG patients (including 31 PACG eyes and 26 PAC/PACS eyes) and normal control eyes.

	Invariable		Multivariable		
	β	*P*	Direction	β	*P*
**Sex**	-0.023	0.559			
**Age (years)**	-0.002	0.439			
**IOP (mmHg)**	0.001	0.727			
**AL (mm)**	0.037	0.069			
**Extent of PAS (clock-hours)**	-0.007	0.172			
**ACA (mm**^**2**^**)**	0.026	**<0.001**		0.013	0.098
**LV (mm)**	-0.292	**<0.001**		0.116	0.305
**LT (mm)**	-0.132	**0.004**		-0.055	0.301
**IC (mm)**	-1.519	**<0.001**	Eyes with smaller IC in the dark condition exhibited greater light-to-dark change in I-area	-1.244	**<0.001**
**IT500μm (mm)**	1.222	**<0.001**		0.209	0.501
**PD (mm)**	0.104	**<0.001**		0.012	0.599
**Diagnostic groups 1**	-0.087	0.041		0.087	0.589
**Diagnostic groups 2**	-0.212	**<0.001**	Compared to control eyes, PACG eyes exhibited smaller light-to-dark change in I-area	-0.115	**0.042**

**Diagnostic groups 1:** PAC/PACS eyes vs normal control eyes

**Diagnostic groups 2:** PACG eyes vs normal control eyes

**I-area:** Iris cross-sectional area

**IOP:** Intraocular pressure

**AL:** Axial length

**PAS:** Peripheral anterior synechiae

**ACA:** Anterior chamber cross-sectional area

**LV:** Lens vault

**LT:** Lens thickness

**IC:** Iris curvature

**IT500μm:** Iris thickness 500μm from the iris root

**PD**: Pupil diameter

## Discussion

Recent studies suggest that both anatomic and physiological factors contribute to the pathogenesis of primary angle closure glaucoma [16]. In addition to anatomic factors, dynamic changes of the iris are implicated in disease pathogenesis [17,18]. However, most studies on dynamic changes of the anterior segment in PACG have compared patients to healthy normal [7,8,19], which may reveal little about disease progression. Here we focused on the asymmetric onset of chronic PACG by comparing changes in the anterior segment of CPACG eyes to fellow PACS/PAC eyes. In our previous study, shallower ACD, shorter AOD500, and thinner IT as measured by UBM were found in PACG eyes compared to fellow PAC/PACS eyes [13, and similar findings were demonstrated in the current study by AS-OCT in light and/or dark conditions. Smaller light-to-dark changes in anterior chamber angle, pupil diameter, and iris area were found in PACG eyes compared to their fellow PAC/PACS eyes. In addition, smaller light-to-dark changes in pupil diameter were found in fellow PAC/PACS eyes compared to normal healthy eyes. The dynamic change of iris area was smallest in CPACG eyes, and largest in normal eyes, and was related to larger iris curvature in CPACG eyes. We suggest that blunted dynamic changes in the anterior chamber may contribute to disease progression.

A previous study on dynamic changes in anterior chamber depth and area found no significant difference between PACG eyes and open-angle eyes [7], while others found smaller light-to-dark AOD500 changes in PAC/PACS eyes than normal eyes [9,20], but no difference among PACS, PAC, and PACG eyes [10,20]. In the current study, AOD500μm changes in CPACG eyes were much smaller than in fellow PACS/PAC eyes. Given that no significant differences in light-to-dark lens parameter changes were found among normal, PAC, and PACG eyes in two previous studies [7,10] as well as the current study, the iris is likely to be responsible for most, if not all, of the dynamic changes in the anterior chamber.

Previous studies found no difference in pupil diameter changes between normal and PACS eyes [19], or between PACG/PAC eyes and fellow eyes with acute PAC during light-to-dark transition [21]. In the current study, however, the pupil diameter change was largest in normal eyes and smallest in CPACG eyes. In PACG eyes and fellow PACS/PAC eyes, this paradoxical pupillary response to light may be attributed to optic nerve damage and contraction−relaxation dysfunction of the iris. *In vivo* measurements of the iris may provide clues on the mechanisms underlying this deficit. In our previous study, CPACG eyes had thinner irises than fellow PAC/PACS eyes [13]. In the current study, iris area was smallest in PACG eyes and largest in normal eyes in the light condition, consistent with a previous study where PACG eyes had thinner irises than normal subjects [22]. These anatomic changes may contribute to contraction−relaxation dysfunction of the iris in CPAGC. In CPACG eyes, a smaller change in iris area between light and dark conditions implies a larger pupil diameter under bright light and less relative dilation from light to dark. Thus, it is not surprising that there is less change in the iris.

In the current study, there were no differences in pupil diameter and iris area between normal eyes and CPACG eyes under the dark condition. Neither iris thickness at 500μm nor at 750μm from the iris root differed between normal eyes and CPACG eyes in the dark condition. Apart from angle crowding, changes in iris configuration in response to light may play an important role in angle closure. It is thought that the pressure difference between anterior and posterior chambers causes forward bowing of the iris, resulting in greater curvature [23]. In the current study, smaller iris curvature in normal eyes strongly suggests that the extent of iris curvature may be an indicator of pupillary block. Moreover, for the first time, we report that eyes with less iris curvature under dark conditions exhibit a greater light-to-dark change in iris area.

The iris is a sponge [18], and dynamic changes in iris area reflect its ability to release fluid from the stroma [24], which is associated with occludable angle [25]. We found that a smaller dynamic change in iris area was correlated with smaller anterior chamber area, a thinner iris, and larger iris curvature. These results are in accord with previous findings that a smaller iris area decrease was associated with shorter axial length in both normal and PACS eyes [19], and that iris volume decrease was associated with a thinner iris in fellow eyes of acute PAC and PAC/PACG eyes [22]. In smaller eyes with shorter axial lengths and smaller anterior chamber areas, the release of fluids from the iris stroma may be limited. Anterior bulk and thinner iris may also affect dynamic changes of the iris.

In this cross-sectional study, reduced iris thickness, iris area, and contraction−relaxation function with PACG progression and severity may be related to iris atrophy owing to chronic elevated intraocular pressure or to aberrant dynamic behavior of the iris. However, a previous study found that eyes with primary open angle glaucoma (POAG), which are also exposed to chronically elevated intraocular pressure, exhibit iris thickness and light response similar to PACS eyes [10]. Moreover, light-to-dark changes in iris area of PAC/PACS eyes were smaller than in POAG eyes [24]. Thus, it appears that chronically elevated intraocular pressure contributes little to the dynamic behavior of the iris.

There are several limitations to this study. 1) The cross-sectional nature of the study design limits exploration of causal associations between disease course and anterior chamber features. 2) The measurements were from horizontal cross-sectional images only and may not be representative of the entire anterior segment. 3) PACG eyes were still treated with topical IOP-reducing medications (other than prostaglandin analogs or osmotic agents) during the study. However, topical cholinergic agents were discontinued for at least 7 days, which should have minimized effects on anterior segment measurements. 4) All of the AS-OCT measurements were performed by a single physician. However, intraobserver reproducibility was acceptable.

In summary, we compared several anterior segment parameters and their dynamic changes from light to dark in CPACG eyes, PAC/PACS fellow eyes, and normal eyes. Dynamic changes of iris area and pupil diameter were smallest in CPACG eyes and largest in normal eyes, a difference attributable to smaller anterior chamber area, thinner iris, and greater iris curvature in CPACG eyes. Although this cross-sectional comparison study does not prove causation, the data strongly suggest that iris dynamics contribute substantially to the progression of PAC to PACG.

## Supporting information

S1 Table Intraobserver repeatability of anterior segment parameters in 60 recruited eyes. (PDF)

S1 File Original data. (XLSX)
