# Supplementary material for: Dynamic changes of anterior segment in patients with different stages of primary angle-closure in both eyes and normal subjects
Source: PLoS One. 2017 May 18;12(5):e0177769. doi: 10.1371/journal.pone.0177769 (PMC5436810; doi:10.1371/journal.pone.0177769)
Supplement: S1 Table — (PDF) [file pone.0177769.s001.pdf]

**S1 Table. Intraobserver repeatability of anterior segment parameters in 60 recruited eyes.**

| | First Measurement | Second Measurement | *P** | ICC | 95% LoA |
|---|---|---|---|---|---|
| **Anterior chamber parameters** | | | | | |
| ACD (mm) | 2.198±0.328 | 2.208±0.221 | 0.356 | 0.996 | -0.084~0.079 |
| ACW (mm) | 11.421±0.467 | 11.211±0.358 | 0.274 | 0.980 | -0.392~0.496 |
| ACA ($mm^2$) | 15.102±3.264 | 15.052±3.230 | 0.241 | 0.992 | -0.749~0.850 |
| **Lens parameters** | | | | | |
| LV (mm) | 0.656±0.286 | 0.669±0.387 | 0.673 | 0.998 | -0.063~0.059 |
| LT (mm) | 4.883±0.383 | 4.712±0.432 | 0.659 | 0.951 | -0.344~0.487 |
| **Anterior chamber angle parameters** | | | | | |
| NAOD500μm (mm) | 0.186±0.133 | 0.155±0.161 | 0.378 | 0.886 | -0.085~0.099 |
| TAOD500μm (mm) | 0.160±0.123 | 0.171±0.230 | 0.296 | 0.890 | -0.074~0.061 |
| NARA750μm ($mm^2$) | 0.049±0.038 | 0.040±0.029 | 0.100 | 0.831 | -0.030~0.047 |
| TARA750μm ($mm^2$) | 0.039±0.039 | 0.036±0.036 | 0.166 | 0.868 | -0.035~0.041 |
| **Iris parameters** | | | | | |
| PD (mm) | 3.720±1.111 | 3.717±1.071 | 0.356 | 0.991 | -0.039~0.042 |
| NIT500μm (mm) | 0.418±0.083 | 0.409±0.101 | 0.785 | 0.991 | -0.088~0.098 |
| TIT500μm (mm) | 0.432±0.084 | 0.440±0.075 | 0.569 | 0.995 | -0.087~0.069 |
| NIT750μm (mm) | 0.431±0.088 | 0.427±0.092 | 0.298 | 0.945 | -0.088~0.069 |
| TIT750μm (mm) | 0.450±0.093 | 0.469±0.085 | 0.389 | 0.967 | -0.099~0.058 |
| NIT1000μm (mm) | 0.442±0.090 | 0.431±0.072 | 0.527 | 0.933 | -0.092~0.107 |
| TIT1000μm (mm) | 0.462±0.094 | 0.449±0.089 | 0.298 | 0.924 | -0.085~0.112 |
| NIC (mm) | 0.287±0.084 | 0.284±0.084 | 0.455 | 0.899 | -0.009~0.012 |
| TIC (mm) | 0.242±0.076 | 0.240±0.058 | 0.742 | 0.906 | -0.011~0.014 |

| | | | | | |
|---|---|---|---|---|---|
| NI-area ($mm^2$) | 1.804±0.243 | 1.792±0.240 | 0.266 | 0.906 | -0.193~0.217 |
| TI-area ($mm^2$) | 1.715±0.232 | 1.700±0.225 | 0.178 | 0.890 | -0.194~0.225 |

***** Paired *t*-test

**ACD:** Anterior chamber depth

**ACW:** Anterior chamber width

**ACA:** Anterior chamber cross-sectional area

**LV:** Lens vault

**LT:** Lens thickness

**NAOD500µm:** Angle opening distance 500µm from the scleral spur on nasal side

**TAOD500µm:** Angle opening distance 500µm from the scleral spur on temporal side

**NARA750µm:** Angle recess area 750µm from the scleral spur on nasal side

**TARA750µm:** Angle recess area 750µm from the scleral spur on temporal side

**PD**: Pupil diameter

**NIT500µm:** Iris thickness 500µm from the iris root on nasal side

**TIT500µm:** Iris thickness 500µm from the iris root on temporal side

**NIT750µm:** Iris thickness 750µm from the iris root on nasal side

**TIT750µm:** Iris thickness750µm from the iris root on temporal side

**NIT 1000µm:** Iris thickness 1000µm from the iris root on nasal side

**TIT 1000µm:** Iris thickness 1000µm from the iris root on temporal side

**NIC:** Iris curvature on nasal side

**TIC:** Iris curvature on temporal side

**NI-area:** Iris cross-sectional area on nasal side

**TI-area:** Iris cross-sectional area on temporal side
